# Changes in Child Undernutrition and Overweight in India From 2006 to 2021: An Ecological Analysis of 36 States

**DOI:** 10.9745/GHSP-D-21-00569

**Published:** 2022-10-31

**Authors:** Jithin Sam Varghese, Aashish Gupta, Rukshan Mehta, Aryeh D. Stein, Shivani A. Patel

**Affiliations:** a Nutrition and Health Sciences Program, Laney Graduate School, Emory University, Atlanta, GA, USA.; b Population Studies Center, University of Pennsylvania, Philadelphia, PA, USA.; c The Hospital for Sick Children (SickKids), Toronto, Canada.; d Hubert Department of Global Health, Emory University, Atlanta, GA, USA.

## Abstract

**Objectives:**

We evaluated changes in priority indicators of child growth from 2006 to 2021 and examined the role of human development measures in these changes.

**Methods:**

We estimated cumulative and annualized changes in state- and district-level child growth indicators using 3 rounds of National Family Health Surveys (2005–2006, 2015–2016, 2019–2021) in 36 states. Outcomes included stunting, underweight, wasting, and overweight. Human development was measured using a principal components analysis of 9 ecological indicators. We contrasted expected versus observed changes in district-level growth outcomes between 2016 and 2021 based on changes in development indicators using 2-way Blinder Oaxaca decomposition.

**Results:**

From 2006 to 2021, the prevalence of stunting, underweight, and wasting decreased by 12.3, 10.3, and 0.7 percentage points, respectively, while the prevalence of overweight increased by 1.9 percentage points. The annualized rate of within-state change for stunting was lower from 2016 to 2021 compared with the 2006 to 2016 period, while the rate of change in overweight was higher. Simultaneously, all 9 human development indicators improved between 2006 and 2021. A unit increase between 2016 and 2021 in the human development score predicted a −5.1 percentage point (95% confidence interval=−5.8, −4.4) change in stunting, yet observed stunting declined by just -2.5 percentage points.

**Conclusions:**

From 2016 to 2021, population-level reduction in child stunting has slowed and the rise in child overweight has accelerated, relative to the 10 years preceding this period.

## Abstract (precis)

India has historically displayed high levels of child stunting and low levels of child overweight. Despite improvements in human development indicators between 2006 and 2021, population-level reductions in child stunting have slowed and child overweight is rising faster than predicted by human development indicators.

## Teaser

**Key Messages:**

- Historically, India has displayed high levels of child stunting and low levels of child overweight. Between 2006 and 2016, improvements in the reduction of child stunting and stability of overweight were observed, but, between 2016 and 2021, those trends have slowed or stagnated and the increase in child overweight has accelerated.
- The slowing progress in child growth indicators occurred despite improvements from 2006 to 2021 in human development indicators—including literacy, sanitation, and households with electricity—that predicted large reductions in stunting and underweight.

**Key Implication:**

- Policy makers and national stakeholders should focus on inter-ministerial efforts to improve human development indicators, while avoiding reversal of gains made in reducing undernutrition and monitoring the simultaneous rise in child overnutrition.

## INTRODUCTION

Since the first cross-national studies of child growth, India has consistently ranked among the countries with the highest levels of growth faltering.^1^^–^^4^ India’s pernicious child undernutrition problem has been attributed to a complex interaction among structural factors such as inadequate food systems; poor water, sanitation, and hygiene infrastructure; and household poverty, which ultimately determine individual dietary intake and morbidities that impact child nutrition.^5^^,^^6^ Since 2005, several ambitious national-level development programs have targeted many of these structural factors through income, education, sanitation, and direct nutrition campaigns. Between 2006 and 2016, nationwide child stunting decreased by 10 percentage points while child overweight increased.^7^^,^^8^ These shifts in undernutrition and overweight occurred across the socioeconomic spectrum.^7^^,^^8^

Between 2015 and 2019, several new national-level campaigns were launched to improve living conditions such as sanitation (Swachh Bharat Mission), clean cooking fuel (Pradhan Mantri Ujjwala Yojana), electrification, and health (Ayushman Bharat-PMJAY).^9^ Simultaneously, the Indian economy experienced shocks such as currency demonetization (November 2016), which worsened the slower economic growth of the 2010s relative to the previous decade and may have adversely affected some districts more than others.^10^ Whether and how this dynamic development policy landscape between 2015 to 2019 impacted trends in child undernutrition and child overweight is not clear.

We conducted state- and district-level analyses of nationally representative surveys of children to evaluate changes in child growth indicators across survey rounds conducted in 36 states and union territories from 2005 to 2006, 2015 to 2016, and 2019 to 2021. As a secondary objective, we assessed whether reductions in undernutrition and overweight predicted by changes in human development indicators were achieved from 2015–2016 to 2019–2021.

We conducted state- and district-level analyses of nationally representative surveys of children to evaluate changes in child growth indicators across 36 states and union territories.

## METHODS

We analyzed data from 3 cross-sectional surveys conducted over 15 years in India. We tracked changes in child undernutrition, overweight, and household development indicators. Some of the indicators listed are used in monitoring India’s progress toward achieving the Sustainable Development Goals.^11^

### Data Sources

We used individual-level data from the National Family Health Surveys (NFHS) conducted from 2005 to 2006 (Round 3), 2015 to 2016 (Round 4), and 2019 to 2021 (Round 5). We refer to the NFHS-3, NFHS-4, and NFHS-5 surveys as 2006, 2016, and 2021, respectively, corresponding to the last year of data collection. NFHS-4 and NFHS-5 employed a multistage sample survey design to generate samples that are representative at the district, state, and national levels for children aged younger than 5 years and adults (women aged 15–49 years, men aged 15–54 years). NFHS-3 was a multistage sample survey that represented state and national levels. Rural villages or urban census enumeration blocks served as sampling clusters, from which households were randomly sampled. NFHS-3 (November 2005–August 2006) surveyed 109,041 households in 28 states and Delhi.^12^ NFHS-4 (January 2015–December 2016) surveyed 601,509 households in 29 states, 7 union territories, and 640 districts.^13^ Phase 1 (June 2019–March 2020) of NFHS-5 surveyed 307,554 households in 17 states, 5 union territories, and 341 districts before the first wave of coronavirus disease (COVID-19).^14^ Phase 2 (November 2020–April 2021) of NFHS-5 surveyed 329,145 households in 11 states, 3 union territories, and 366 districts. Additional information on changes in geographic boundaries between rounds is provided in Note 1 and Figure 1 in Supplement 1. We used shapefiles provided by the Demographic and Health Surveys program (www.dhsprogram.com) to classify NFHS-5 primary sampling units into NFHS-4 districts.

### Child Growth Outcomes

State- and district-level prevalence of child growth indicators were treated as the outcomes. All outcomes are expressed as the percentage of children aged younger than 5 years exhibiting the indicator. Stunting was defined as height-for-age z-score < −2 standard deviations (SD) from the median based on World Health Organization growth standards. Underweight was defined as weight-for-age z-score < −2 SD.^15^ Wasting was defined as weight-for-height z-score < −2 SD.^15^ Overweight or obesity in children was defined as weight-for-height z-score > 2 SD.^15^ We excluded those children with extreme or unavailable values for any of height-for-age z-score (<−6 or >6), weight-for-age z-score (<−6 or >5), and weight-for-height z-score (<−5 or >5) as per World Health Organization standards. Additional information on measurement protocol and quality control is provided in Note 2 of Supplement 1.

### Human Development Indicators

We focus on 2 domains of human development: human capital and standard of living. Human capital was measured by 4 indicators (percentage of women who are literate, percentage of women with 10 or more years of schooling, percentage of female children at birth, and percentage of women aged 20–24 years who married or first cohabited at age 18 or later), and standard of living was measured by 5 indicators (households with improved sanitation, improved drinking water, electricity, clean cooking fuel, or covered by any health insurance scheme).

### Statistical Analysis

We conducted an ecological analysis of survey-weighted estimates derived from individual-level data of child malnutrition and development indicators for 36 states and 640 districts (2015 boundaries) at the state and district levels, respectively. We estimated annualized state-level change between surveys as average absolute change in percentage points per year. We tested for difference in state-level annualized change between 2006 and 2016 and 2016 and 2021 using a paired Wilcoxon signed-rank test. We also estimated district-level changes in growth indicators between 2016 and 2021.

We benchmarked changes in growth indicators according to targets set by POSHAN Abhiyaan (National Nutrition Mission), India’s nationally coordinated flagship nutrition campaign launched in March 2018.^16^^,^^17^ POSHAN Abhiyaan announced targets to reduce stunting and underweight by 2 percentage points per year between 2019 and 2022.^18^^,^^19^ In the absence of a national target, we assumed a target of 2 percentage points per year for wasting.

In addition, we examined expected changes in child growth indicators from 2016 to 2021 based on changes in the set of human development indicators by estimating a 2-way Blinder-Oaxaca decomposition (unweighted, bivariate). In the bivariate decomposition, the expected change reflects the change in the child growth indicator that is predicted (“explained”) by the human development indicator. The difference between the expected and observed changes reflects the change in the nutrition outcome that was not predicted (“unexplained”) by the human development indicator. For example, a smaller observed reduction in an undernutrition indicator relative to the expected reduction suggests unrealized improvements that were anticipated based on secular trends predicted by changes in human development. Our primary measure of human development was a weighted composite score of 9 indicators of district-level human development that was derived using principal component analysis of these indicators. In addition, we report the decomposition findings for each of the human development indicators individually. All analysis was conducted using R version 3.6.1 and Stata version 13.

### Ethical Approval

The study was determined to be human subjects exempt research by the Emory University Institutional Review Board.

## RESULTS

### Child Growth Outcomes

From 2006 to 2021, the population-weighted prevalence of stunting declined from 47.8% to 35.5% (12.3 percentage point reduction) and that of underweight declined from 42.4% to 32.1% (10.3 percentage point reduction) (Table 1). Over the same 15-year period, the prevalence of wasting was unchanged (about 20%) and that of overweight increased from 1.5% to 3.4% (1.9 percentage point increase). At the national level, the annualized rate of change in stunting was lower from 2016 to 2021 compared with 2006 to 2016 (−0.6 percentage points per year versus −0.9 percentage points per year, respectively; *P*=.10) (Figures 2 and 3 in Supplement 1). The annualized rate of change in wasting was different (−0.4 percentage points per year from 2016 to 2021 versus 0.1 percentage points per year from 2006 to 2016; *P*<.05) between the 2 study periods. There was no difference in the annual rate of change in underweight (−0.7 percentage points per year versus −0.7 percentage points per year, respectively; *P*=.58) across the 2 study periods at the national level. The annualized rate of change in overweight was higher but not statistically significant at the national level, from 2016 to 2021 compared with 2006 to 2016 (0.1 percentage points per year versus 0.3 percentage points per year, respectively, *P*=.22). Annualized rates of change for stunting and overweight were significantly different between the 2 periods (*P*<.01) at the state level. As of 2021, the prevalence of stunting, wasting, and underweight for the nation exceeded the 2022 POSHAN Abhiyaan targets for stunting, wasting, and underweight by 11 percentage points, 10 percentage points, and 8 percentage points, respectively (Table 1).

**TABLE 1.** Prevalence and Change in State-Level Growth Indicators Among Children Aged Younger Than 5 Years in 36 States in India, 2006–2021^a^

	**2006 Prevalence, %** **(95% CI)**	**2016** **Prevalence, %** **(95% CI)**	**2021 Prevalence, %** **(95% CI)**	**2006–2016**^b^ **Annualized Change, PP** **(95% CI)**	**2016–2021**^b^ **Annualized Change, PP** **(95% CI)**	***P* Value for Overall Difference in Annualized Change** ^c^	***P* Value for Within-State Difference in Annualized Change**^c^ **(n=30)**	**Poshan Abhiyaan Target for 2022** ^d^
All India								
Stunting	47.8 (47, 48.7)	38.4 (38, 38.7)	35.5 (35.1, 35.8)	−0.9 (−1.0, −0.9)	−0.6 (−0.7, −0.5)	.10	<.01	26.4
Wasting	20.0 (19.4, 20.6)	21.1 (20.8, 21.4)	19.3 (19, 19.6)	0.1 (0, 0.2)	−0.4 (−0.4, −0.3)	<.05	.17	9.1
Underweight	42.4 (41.6, 43.3)	35.7 (35.4, 36.1)	32.1 (31.8, 32.5)	−0.7 (−0.8, −0.6)	−0.7 (−0.8, −0.6)	.42	.09	23.7
Overweight	1.5 (1.4, 1.7)	2.1 (2.0, 2.2)	3.4 (3.3, 3.6)	0.1 (0.0, 0.1)	0.3 (0.2, 0.3)	.22	<.01	-
Rural								
Stunting	50.5 (49.6, 51.5)	41.1 (40.8, 41.5)	37.3 (36.9, 37.7)	−0.9 (−1, −0.8)	−0.8 (−0.9, −0.7)	.27	<.01	29.1
Wasting	20.9 (20.2, 21.6)	21.5 (21.2, 21.8)	19.5 (19.1, 19.9)	0.1 (0, 0.1)	−0.4 (−0.5, −0.3)	.06	.56	9.5
Underweight	45.6 (44.6, 46.5)	38.2 (37.9, 38.6)	33.8 (33.4, 34.1)	−0.7 (−0.8, −0.6)	−0.9 (−1.0, −0.8)	.29	.02	26.2
Overweight	1.2 (1.1, 1.4)	1.8 (1.7, 1.9)	3.2 (3.1, 3.3)	0.1 (0, 0.1)	0.3 (0.2, 0.3)	−.23	<.01	-
Urban								
Stunting	39.5 (37.9, 41.0)	31.0 (30.3, 31.8)	30.1 (29.3, 30.9)	−0.8 (−1, −0.7)	−0.2 (−0.4, 0)	.01	.01	19
Wasting	17.0 (16.0, 18.1)	20.0 (19.4, 20.7)	18.6 (18.0, 19.2)	0.3 (0.2, 0.4)	−0.3 (−0.5, −0.1)	.02	.16	8
Underweight	32.7 (31.2, 34.2)	29.1 (28.4, 29.8)	27.4 (26.6, 28.1)	−0.4 (−0.5, −0.2)	−0.4 (−0.6, −0.1)	.49	.79	17.1
Overweight	2.6 (2.1, 3.0)	2.8 (2.5, 3.0)	4.2 (3.9, 4.5)	0 (0, 0.1)	0.3 (0.2, 0.4)	.17	<.01	-

Abbreviations: CI, confidence interval; NFHS, National Family Health Survey; PP, percentage points; SD, standard deviation.

^a^Values are survey-weighted estimates and 95% confidence intervals. Data for 2006, 2016, and 2021 come from NFHS-3, NFHS-4, and NFHS-5, respectively. Child stunting was defined as height-for-age < −2SD, child wasting was defined as weight-for-height < −2SD, child underweight was defined as weight-for-age < −2SD, and child overweight was defined as weight-for-height > 2SD.

^b^Annualized change is mean annual absolute change in prevalence for 36 states reported by NFHS surveys in percentage as (%) per annum.

^c^The overall difference for 2006–2016 and 2006–2021 annualized change estimates was tested using z-tests. Within-state differences for 2006–2016 and 2006–2021 annualized change estimates were tested using paired Wilcoxon signed-rank test for 30 states which were available across rounds; Andhra Pradesh values used for Telangana.

^d^POSHAN Abhiyaan target is estimated as prevalence in 2016 minus targeted annual reduction of 2% for stunting and underweight in children. We additionally used a target of 2 percentage points per year for wasting.

Note: Additional details on geographical boundaries are presented in Supplement 1 Note 1.

At the state level in rural settings, we observed slowing reductions (mean difference in annualized rates) in stunting (0.8 percentage points per year, *P*<.01) and underweight (0.6 percentage points per year, *P*=.02), and faster increases in overweight (0.3 percentage points per year, *P*<.01) comparing the annualized rates from the 2016 to 2021 period with 2006 to 2016. In urban settings, consistent with national trends, we observed slowing reductions in stunting (0.7 percentage points per year, *P*<.01), similar reductions in underweight (0.1 percentage points per year, *P*=.79), and an accelerating rise in overweight (0.3 percentage points per year, *P*<.01) between 2016 and 2021, relative to 2006 to 2016.

The Figure displays district-level changes in growth indicators from 2016 to 2021. Most districts (68%, 60%, and 66%) experienced reductions in stunting, wasting, and underweight, respectively. Far fewer districts (26%, 27%, and 30%, respectively) experienced reductions of 7 percentage points or more (the rate needed to achieve targets) in stunting, wasting, and underweight. Most districts (72%) experienced an increase in overweight. We provide the uncertainty estimates for state and district prevalence of child malnutrition and development indicators in Supplement 2, and the distribution of these changes in Figures 4 and 5 in Supplement 1.

From 2016 to 2021, most districts experienced reductions in stunting, wasting, and underweight and also experienced an increase in overweight.

**FIGURE:**
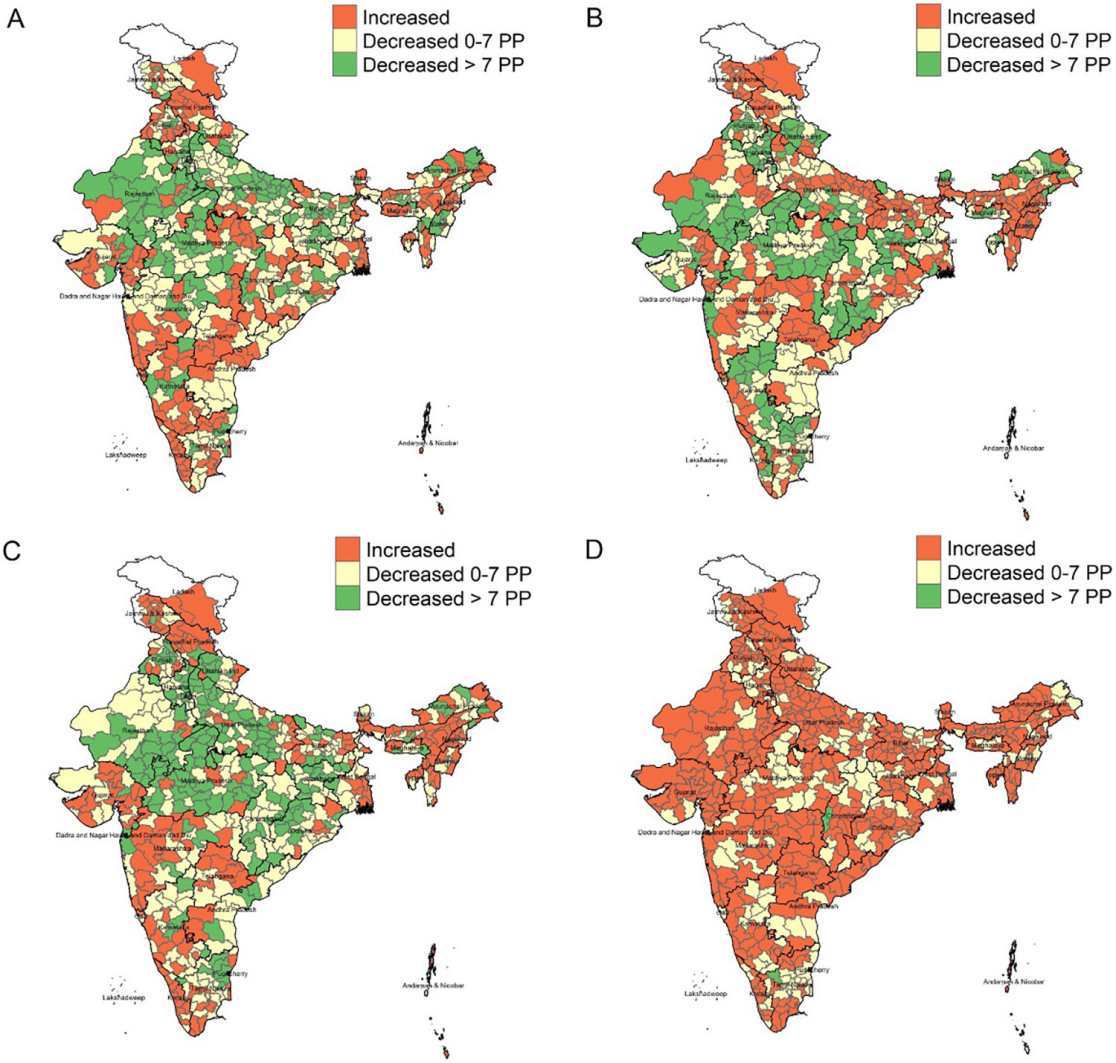
District-Level Changes in Child Growth Indicators in India, 2016–2021: (A) Stunting; (B) Wasting; (C) Underweight; (D) Overweight Abbreviation: PP, percentage points.

Reflecting improvements in development from 2006 to 2021 (Table 2), there were positive state-level absolute annualized rates of change in each of the human development indicators examined over both time periods. After accounting for sampling uncertainty, estimates of national-level annualized change between 2006 and 2016 and 2016 and 2021 were different for only 3 of 9 development indicators. However, paired tests of annualized changes at the state level were different for 6 of 9 indicators, suggesting that there was heterogeneity across state-level annualized changes between the 2 periods. The largest absolute annualized changes from 2016 to 2021 were observed in the percentage of households with improved sanitation facilities (4.3 percentage points per year) and households using clean fuel for cooking (3.0 percentage points per year). The development score summarizing all 9 human development indicators accounted for 47% of the variance in individual indicators. This score increased over survey rounds.

**TABLE 2.** Change in Human Development Indicators in 36 States in India, 2006–2021^a^

	**2006** **Prevalence, %** **(95% CI)**	**2016** **Prevalence, %** **(95% CI)**	**2021** **Prevalence, %** **(95% CI)**	**2006–2016**^b^ **Annualized Change, PP** **(95% CI)**	**2016–2021**^b^ **Annualized Change, PP** **(95% CI)**	***P* Value for Overall Difference in Annualized Change** ^c^	***P* Value for Within-State Difference in Annualized Change** ^c^ **(n=30)**
Women who are literate	55.1 (54.1, 56.1)	68.4 (68.1, 68.7)	71.5 (71.2, 71.7)	1.3 (1.2, 1.4)	0.6 (0.5, 0.7)	.06	<.01
Women with 10 or more years of schooling	22.3 (21.5, 23.1)	35.7 (35.3, 36.1)	41 (40.7, 41.4)	1.3 (1.2, 1.4)	1.1 (1.0, 1.2)	.28	.6
Women aged 20–24 years first cohabiting after age 18 years	55.5 (54.3, 56.7)	74.7 (74.3, 75.2)	77.7 (77.3, 78.1)	1.9 (1.8, 2.1)	0.6 (0.5, 0.7)	<.01	<.01
Female children at birth for children born in last 5 years^d^	47.9 (47.3, 48.4)	47.8 (47.5, 48.0)	48.1 (47.8, 48.3)	0 (−0.1, 0.1)	0.1 (0, 0.1)	.44	.24
Population living in households that use improved sanitation facility	29.5 (28.4, 30.7)	48.5 (48.0, 49.0)	70.2 (69.8, 70.5)	1.9 (1.8, 2.0)	4.3 (4.2, 4.5)	<.01	<.01
Population living in households with improved drinking-water source	88.2 (87.4, 89.0)	94.4 (94.2, 94.6)	95.8 (95.6, 95.9)	0.6 (0.5, 0.7)	0.3 (0.2, 0.3)	.23	<.01
Population living in households with electricity	67.2 (65.9, 68.5)	88.0 (87.7, 88.3)	96.8 (96.7, 96.9)	2.1 (1.9, 2.2)	1.8 (1.7, 1.8)	.25	<.01
Households using clean fuel for cooking	25.5 (24.3, 26.8)	43.9 (43.2, 44.5)	58.8 (58.3, 59.3)	1.8 (1.7, 2.0)	3.0 (2.8, 3.1)	<.01	<.01
Households with any usual member covered under health insurance or financing scheme^e^	5.0 (4.6, 5.4)	28.8 (28.5, 29.2)	41.2 (40.9, 41.5)	2.4 (2.3, 2.4)	2.5 (2.4, 2.6)	.42	.76
Human development composite score^f^		−0.1 (−3.8, 3.8)	1.4 (−1.7, 4.3)				

Abbreviation: CI, confidence interval; PP, percentage points.

^a^Data for 2006, 2016, and 2021 come from NFHS-3, NFHS-4, and NFHS-5, respectively. Additional details on geographical boundaries are available in Supplement 1 Note 1.

^b^Annualized change is mean annual absolute change in prevalence.

^c^The overall difference for 2006–2016 and 2006–2021 annualized change estimates was tested using z-tests. Within-state differences for 2006–2016 and 2006–2021 annualized change estimates were tested using paired Wilcoxon Signed-Rank test for 30 states which were available across rounds; Andhra Pradesh values used for Telangana.

^d^Sex ratio can be derived using this indicator.

^e^Ayushman Bharat-PMJAY was not fully rolled out at time of NFHS-5.

^f^Median (2.5 percentile, 97.5 percentile). Score was created using indicators from NFHS-4 districts as reference.

The modeled differences between the observed and expected district-level changes in child growth indicators associated with changes in human development between 2016 and 2021 are shown in Table 3. Changes in the human development score predicted statistically significant reductions in all 3 measures of undernutrition. The observed reductions fell short of the predictions for stunting and underweight but were larger than predicted for wasting. Taking stunting as an example, given the change in the development score from 2016 to 2021, we would predict a 5.1 percentage point (95% confidence interval [CI]=−5.9, −4.3) reduction in stunting. Instead, the prevalence of stunting changed by just −2.5 percentage points, so that observed levels of stunting were 2.6 percentage points (95% CI=1.8, 3.4) higher than what we would expect based on changes in human development. In contrast to predicted undernutrition outcomes, the human development score predicted a modest increase in overweight (0.6 percentage points; 95% CI=0.4, 0.7). Observed changes in overweight exceeded this prediction by 1.1 percentage points (95% CI=0.8, 1.4).

**TABLE 3.** Expected and Observed District-Level Changes in Child Growth Indicators Associated With Changes in Human Development Indicators, India, 2015–2016 and 2019–2021 (n=640 Districts)

	**Stunting** ^a^		**Wasting** ^a^		**Underweight** ^a^		**Overweight** ^a^	
	**Observed Change −2.5%**		**Observed Change −2.2%**		**Observed Change −3.3%**		**Observed Change 1.7%**	
Observed Percentage Point Change, Mean	**Expected Change, PP** **(95% CI)**	**Difference Between Observed and Expected Change**^b^ **2016–2021, PP (95% CI)**	**Expected Change, PP** **(95% CI)**	**Difference Between Observed and Expected Change**^b^ **2016–2021, PP (95% CI)**	**Expected Change, PP** **(95% CI)**	**Difference Between Observed and Expected Change**^b^ **2016–2021, PP (95% CI)**	**Expected Change, PP** **(95% CI)**	**Difference Between Observed and Expected Change**^b^ **2016–2021, PP (95% CI)**
Mean human development, composite score	−5.1 (−5.9, −4.3)	2.6 (1.8, 3.4)	−1.7 (−2.0, −1.3)	−0.5 (−1.3, 0.3)	−5.5 (−6.3, −4.7)	2.2 (1.2, 3.3)	0.6 (0.4, 0.7)	1.1 (0.8, 1.4)
Percentage of women who are literate	−1.7 (−2.4, −1.0)	−0.9 (−1.8, 0)	−0.6 (−0.8, −0.4)	−1.6 (−2.4, −0.7)	−1.9 (−2.5, −1.2)	−1.4 (−2.4, −0.4)	0.2 (0.1, 0.3)	1.5 (1.2, 1.8)
Percentage of women with 10 or more years of schooling	−2.3 (−3.0, −1.6)	−0.2 (−1.0, 0.5)	−0.8 (−1.0, −0.6)	−1.4 (−2.2, −0.6)	−2.5 (−3.1, −1.9)	−0.8 (−1.7, 0.2)	0.3 (0.2, 0.3)	1.4 (1.1, 1.7)
Percentage of women aged 20–24 years first cohabiting after age 18 years	−0.1 (−0.2, 0)	−2.5 (−3.5, −1.4)	−1.7 (−2.0, −1.3)	−0.5 (−1.3, 0.3)	−0.2 (−0.4, −0.1)	−3 (−4.3, −1.8)	0.1 (0, 0.2)	1.6 (1.3, 1.9)
Mean sex ratio at birth for children born in the last five years (females per 1,000 males)	0 (−0.1, 0)	−2.5 (−3.5, −1.6)	0 (0, 0)	−2.2 (−3.0, −1.4)	0 (−0.1, 0.1)	−3.2 (−4.3, −2.2)	0 (0, 0.1)	1.7 (1.4, 2)
Percentage of population living in households that use an improved sanitation facility	−6.5 (−7.2, −5.7)	3.9 (2.8, 5.0)	−2.9 (−3.5, −2.3)	0.8 (−0.2, 1.7)	−8.2 (−9.1, −7.2)	4.9 (3.8, 6.0)	0.9 (0.7, 1.1)	0.8 (0.4, 1.1)
Percentage of population living in households with an improved drinking-water source	−0.1 (−0.2, 0.1)	−2.5 (−3.5, −1.5)	−0.2 (−0.3, 0)	−2 (−2.8, −1.2)	−0.1 (−0.3, 0.1)	−3.2 (−4.3, −2.0)	0.0 (−0.1, 0.0)	1.7 (1.4, 2.0)
Percentage of population living in households with electricity	−5.2 (−6.3, −4.2)	2.7 (1.6, 3.8)	−1 (−1.6, −0.5)	−1.2 (−2.2, −0.1)	−4.4 (−5.5, −3.4)	1.2 (−0.2, 2.5)	0.3 (0.1, 0.5)	1.4 (1.0, 1.8)
Percentage of households using clean fuel for cooking	−3.1 (−3.8, −2.5)	0.6 (−0.3, 1.5)	−0.9 (−1.2, −0.6)	−1.3 (−2.0, −0.5)	−3.2 (−3.9, −2.6)	−0.1 (−1.1, 1.0)	0.4 (0.3, 0.5)	1.3 (1.0, 1.6)
Percentage of households with any usual member covered under a health insurance or financing scheme	−1.4 (−1.8, −1.0)	−1.1 (−2.2, −0.1)	0 (−0.2, 0.2)	−2.2 (−3.0, −1.3)	−0.8 (−1.3, −0.4)	−2.4 (−3.5, −1.4)	0 (−0.1, 0.1)	1.7 (1.3, 2.0)

Abbreviations: CI, confidence interval; NFHS, National Family Health Survey; PP, percentage points, SD, standard deviation.

^a^Child stunting was defined as height-for-age < −2SD. Child wasting was defined as weight-for-height < −2SD. Child underweight was defined as weight-for-age < −SD. Child overweight was defined as weight-for-height > 2SD. All values are coefficient (bootstrapped 95% confidence interval).

^b^Difference between observed and expected change between 2016 (NFHS-4) and 2021 (NFHS-5). Contributions are modelled through 2-way Blinder-Oaxaca decomposition with each indicator entered separately. Equal weights (w=0.5) were applied for both survey rounds. The decomposition model gives each of the 640 districts analyzed equal weight, and district grand mean will not sum to the state population-weighted mean.

With respect to individual development indicators, 8 of 9 predicted statistically significant reductions in 1 or more child undernutrition indicators and 7 of 9 predicted significant increases in overweight. For many development indicators, reductions in undernutrition were smaller than expected while the rise in overweight was larger than expected.

## DISCUSSION

Comparing the periods 2006–2016 and 2016–2021, the pace of reductions in child stunting and underweight across Indian states has slowed or stagnated whereas the rise of child overweight has accelerated. These changes disrupt favorable trends that included larger reductions in stunting and stability of overweight observed from 2006 to 2016. Our findings also indicate that India is unlikely to achieve the national targets set by POSHAN Abhiyaan for stunting and underweight of 26% and 24%, respectively, by the end of 2022.^17^^,^^20^ While there is no national target for the prevalence of child overweight in 2022, the projected prevalence in 2022 of nearly 4% is closer to the average among low- and middle-income countries in 2017 (6.0%).^21^^–^^23^ Given that the rise in the proportion of overweight children is expected to offset any reductions in undernutrition, the total prevalence of unhealthy weight among children in India will likely remain unchanged.^24^^,^^25^ Mirroring the national scenario, in both rural and urban areas, we found that progress in undernutrition slowed, while overweight increased.

The slowing progress in undernutrition occurred against the backdrop of improvements in most development indicators from 2016 to 2021. Our findings highlight a potentially worrisome disconnect between the apparent expansion of human development and improvements in child nutrition outcomes.^26^ Specifically, the bivariate decomposition analysis predicted moderate to large declines in population-level stunting and wasting based on the rise in favorable development indicators such as women’s literacy, improved sanitation, and households with electricity. Our analyses showed that these predicted reductions in undernutrition did not occur. Moreover, overweight is rising at a rate that is greater than what would be expected by improvements in human development observed in the past 5 years.

Our findings highlight a potentially worrisome disconnect between the apparent expansion of human development and improvements in child nutrition outcomes.

At a national level, we found that the prevalence of stunting decreased by 0.6% between 2016 and 2021, with nearly 15% of districts experiencing a 5 percentage points or larger rise in the prevalence of stunting despite improvements in development indicators. In other settings, the impact of nutrition-sensitive and nutrition-specific interventions on child growth outcomes has been mixed, with some studies reporting reductions^27^^,^^28^ and others reporting no change^29^ in child stunting. The observed slowing declines in child stunting also paralleled a reversal in the decline in annual infant mortality rates,^30^ suggesting alarming trends in nutrition-sensitive child health outcomes. Targeted monitoring of nutrition outcomes and careful investigation of district-level heterogeneity in trends may be informative to develop locally tailored strategies to more effectively address the lingering burden of child stunting, especially in areas that are worsening.

From 2016 to 2021, there was virtually no national-level change in child wasting, a reflection of current deficits in tissue and fat mass. The stagnation in wasting may signal entrenched insufficient access to diets required for optimal growth among children at the population level. It must be noted, however, that the prevalence of wasting is sensitive to seasonal variations in food availability and infectious disease.^31^ Given the differential timing of data collection across NFHS survey rounds, it is possible that the observed stagnation in prevalent wasting is an artifact of seasonality.

The rapid rise in child overweight presents a challenge to India. Cohort studies from low- and middle-income countries suggest that childhood overweight persists into adulthood,^32^^,^^33^ with implications for risk of diabetes and cardiovascular disease. Moreover, South Asians as a group are at higher risk for cardiometabolic disorders at earlier ages and lower body mass index.^34^ Even in children aged 5 to 19 years, metabolic abnormalities such as high triglycerides are present at all levels of weight status, with progressively higher levels of metabolic morbidities with increasing body mass index-for-age.^26^ Rising overweight in childhood combined with the elevated risk of metabolic diseases in ethnic South Asians creates a perfect storm for higher morbidity, mortality, and medical costs due to cardiometabolic disease over the life course.

Rising overweight in childhood combined with the elevated risk of metabolic diseases in ethnic South Asians create a perfect storm for higher morbidity, mortality, and medical costs due to cardiometabolic disease over the life course.

### Strengths and Limitations

Overall, our analysis has several strengths. We used the latest available data to evaluate and benchmark ongoing efforts to reduce child malnutrition in India and set priorities for global reductions in child undernutrition. The application of decomposition analysis permitted us to explore the hypothetical reductions that should have been achieved based on improvements in human development indicators in the population. However, our analyses must be interpreted in light of data limitations. NFHS-5 data was collected partly after the first wave of COVID-19 lockdowns in 14 states. The present ecological analysis did not separate the trends in child health due to economic changes before the pandemic and trends due to the disruption to the economy and society during the lockdowns from the first wave of COVID-19. Restricting our analysis to the 22 states and union territories that included only pre-pandemic data, we found similar patterns for stunting, wasting, and overweight, and slowing of reductions of underweight (data not shown). In addition, precision of outcome estimates for the ecological analysis varied by survey round, as seen for NFHS-3 and NFHS-4 (Supplement 2). We did not explore the association of changes in micronutrient supplementation and of provision of supplementary nutrition with changes in malnutrition outcomes since this was beyond the scope of our analysis.^35^ Finally, our decomposition analysis described associations between human development indicators and child nutrition measured over the same period. In doing so, we were not able to evaluate a lag between human development and improvements in child undernutrition.

## CONCLUSIONS

In light of the unprecedented economic and psychosocial strain resulting from the COVID-19 pandemic, attention to child nutrition will be even more important in the coming years.^36^^,^^37^ India’s economy shrunk for the first time in between the periods of NFHS-5 data collection due to the nationwide lockdowns. As a result, many nutrition-sensitive programs were affected, which may have resulted in a disruption of the trend that we were reporting on and may have contributed to higher undernutrition. Projections show that in the absence of additional social safety net programs to tackle mounting food insecurity and financial distress incurred through the COVID-19–related economic shutdowns, there may be a doubling of the number of wasted and stunted children in South Asia alone by 2022.^38^ Groups with the highest burden of child undernutrition, such as the urban and rural poor, also tended to be the most vulnerable to pandemic-related disruptions.^39^ In these circumstances, careful investigation of the impacts of the pandemic on nutritional indicators is an urgent priority, with a particular focus on the most marginalized and vulnerable.^40^^,^^41^

In summary, the observed slowing progress in measures of child undernutrition warrants further exploration when considered against apparent indicators of progress. Further investigation is prudent to determine whether inequitable human development across segments of the population or broader social and economic shocks—such as demonetization or the COVID-19 pandemic-related disruptions—contribute to slowing rates of reductions in undernutrition. Furthermore, investigation of outcomes beyond anthropometric growth, such as cognition, would provide a more comprehensive picture of the impact of changes in socioeconomic conditions on child social and developmental outcomes.

## Supplementary Material

21-00569-Patel-Supplement-2.xlsx

21-00569-Patel-Supplement-1-clean.pdf

## Notes

**Peer Reviewed**

**First published online:** September 19, 2022.
