# Supplementary material for: Changes in Child Undernutrition and Overweight in India From 2006 to 2021: An Ecological Analysis of 36 States
Source: Glob Health Sci Pract. 2022 Oct 31;10(5):e2100569. doi: 10.9745/GHSP-D-21-00569 (PMC9622276; doi:10.9745/GHSP-D-21-00569)
Supplement: 21-00569-Patel-Supplement-1-clean.pdf [file 21-00569-Patel-Supplement-1-clean.pdf]

Supplement to: Varghese JS, Gupta A, Mehta R, Stein AD, Patel SA. Changes in child undernutrition and overweight in India from 2006 to 2019: an ecological analysis of 36 states. *Glob Health Sci Pract*. 2022;10(5):e2100569. https://doi.org/10.9745/GHSP-D-21-00569

Contents

Supplement Note 1. Technical Note on state and district boundaries 2

Supplement Note 2. Protocol for anthropometric assessment 3

Supplement Figure 1. Flowchart of states and districts included in National Family Health Surveys (2005-2020)... 4

Supplement Figure 2. Scatterplot of annualized changes at state-level between 2006-16 and 2016-21 for child malnutrition (n = 30) 5

Supplement Figure 3. Distribution of annualized change of child malnutrition (n = 30)............... 6

Supplement Figure 4. Scatterplot of annualized changes at state-level between 2006-16 and 2016-21 for development indicators (n = 30) 7

Supplement Figure 5. Distribution of annualized change for development indicators (n = 30)..... 8

Supplement to: Varghese JS, Gupta A, Mehta R, Stein AD, Patel SA. Changes in child undernutrition and overweight in India from 2006 to 2019: an ecological analysis of 36 states. *Glob Health Sci Pract*. 2022;10(5):e2100569. https://doi.org/10.9745/GHSP-D-21-00569

Supplement Note 1. Technical Note on state and district boundaries

Jammu & Kashmir and Ladakh: In October 2019, a former state Jammu & Kashmir (J&K) was separated into a union territory of the same name and Ladakh (comprising of two districts – Leh and Kargil). NFHS-5 reports estimates for J&K and Ladakh separately. For our state-level analysis using NFHS-4 data, we create survey-weighted estimates separately for Jammu & Kashmir and Ladakh.
Daman & Diu and Dadra & Nagar Haveli*:* Two union territories Daman & Diu and Dadra & Nagar Haveli which were previously separate have now been combined, and are reported as such. NFHS-5 reports a combined estimate for Daman & Diu and Dadra & Nagar Haveli. We create NFHS-4 estimates for Daman & Diu and Dadra & Nagar Haveli using survey weights provided.
Andhra Pradesh and Telangana*:* The state of Telangana was formed from Andhra Pradesh in June 2014. We use estimates for Andhra Pradesh in NFHS-3 for both states to calculate annualized changes.
***Districts from existing districts:* Of the 341 districts reporting aggregate data in NFHS-5, 73 were formed from 36 districts by splitting or joining their administrative boundaries. We used shapefiles provided by the Demographic and Health Surveys program (www.dhsprogram.com) to classify NFHS-5 primary sampling units into NFHS-4 districts.**

Supplement to: Varghese JS, Gupta A, Mehta R, Stein AD, Patel SA. Changes in child undernutrition and overweight in India from 2006 to 2019: an ecological analysis of 36 states. *Glob Health Sci Pract*. 2022;10(5):e2100569. https://doi.org/10.9745/GHSP-D-21-00569

Supplement Note 2. Protocol for anthropometric assessment

Anthropometric and biochemical indicators are measured for children under 6 years (0-71 months) residing within the sampled households after informed consent is obtained for different tests separately. SECA 874 U digital scale is used for weighing children. SECA 417 Infantometer is used for measuring length of children under 2 years or less than 85 cm. SECA 213 Stadiometer is used for measuring height. Detailed information on procedure used for anthropometric and biochemical measurement is available for NFHS-4 (1). Information on quality assurance mechanisms adopted is available for NFHS-5 (2).

References:

1. Clinical Anthropometric Biochemical (CAB) Manual. Mumbai: International Institute for Population Sciences; 2014 December 2014. URL: rchiips.org/NFHS/NFHS4/manual/NFHS-4 Biomarker Field Manual.pdf

2. Administrative Procedures in Survey Implementation and Protocols to Ensure Data Quality. 2018. URL: http://rchiips.org/nfhs/NFHS-5sub_presentation.shtml.

Supplement to: Varghese JS, Gupta A, Mehta R, Stein AD, Patel SA. Changes in child undernutrition and overweight in India from 2006 to 2019: an ecological analysis of 36 states. *Glob Health Sci Pract*. 2022;10(5):e2100569. https://doi.org/10.9745/GHSP-D-21-00569

Supplement Figure 1. Flowchart of states and districts included in National Family Health Surveys (2005-2020)

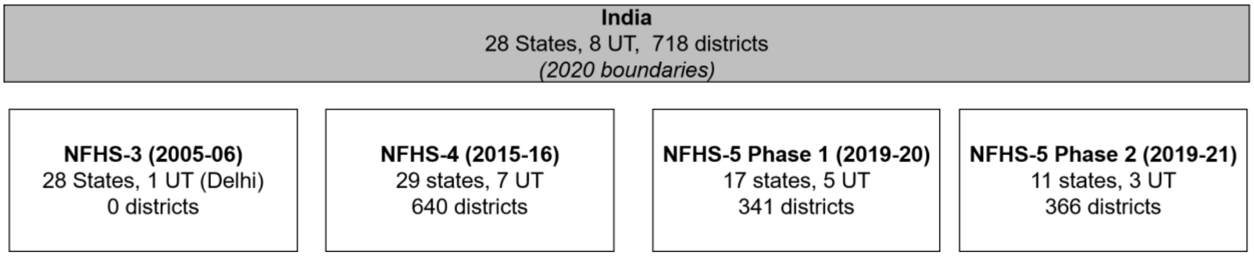

UT: Union territory. UTs are federal territories governed by the Central Government of India based in New Delhi. The list of UTs in 2020 are Andaman & Nicobar Islands, Chandigarh, Dadra & Nagar Haveli and Daman & Diu, Delhi (National Capital Territory), Jammu & Kashmir, Lakshadweep, Ladakh, Puducherry. Additional information on how geographical boundaries changed from 2005 to 2020 are provided in **Supplement Note 1**.

Supplement to: Varghese JS, Gupta A, Mehta R, Stein AD, Patel SA. Changes in child undernutrition and overweight in India from 2006 to 2019: an ecological analysis of 36 states. *Glob Health Sci Pract*. 2022;10(5):e2100569. https://doi.org/10.9745/GHSP-D-21-00569

Supplement Figure 2. Scatterplot of annualized changes at state-level between 2006-16 and 2016-21 for child malnutrition (n = 30)

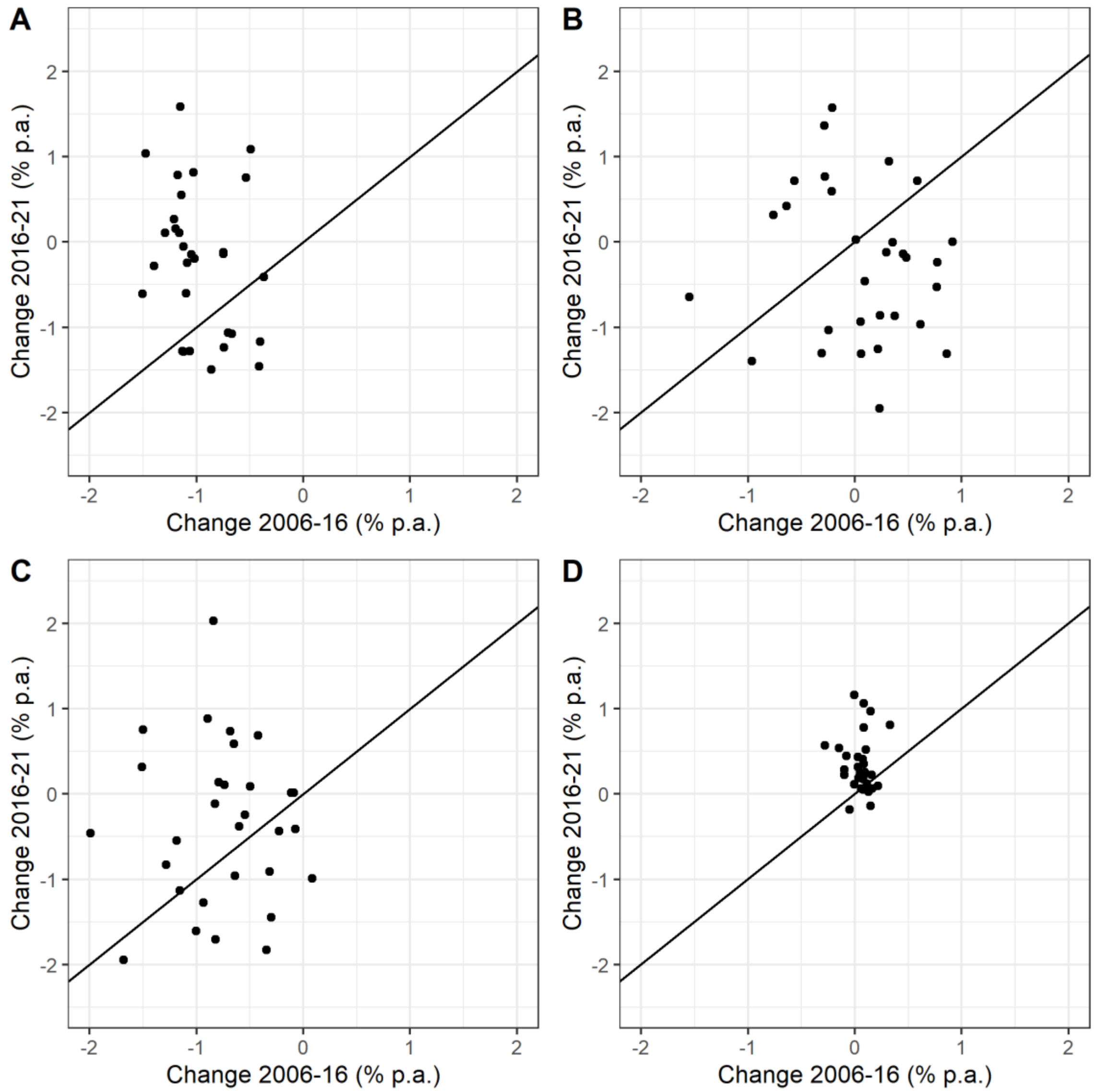

p.a.: percentage points per annum; A: Stunting; B: Wasting; C: Underweight; D: Overweight

Supplement to: Varghese JS, Gupta A, Mehta R, Stein AD, Patel SA. Changes in child undernutrition and overweight in India from 2006 to 2019: an ecological analysis of 36 states. *Glob Health Sci Pract*. 2022;10(5):e2100569. https://doi.org/10.9745/GHSP-D-21-00569

Supplement Figure 3. Distribution of annualized change of child malnutrition (n = 30)

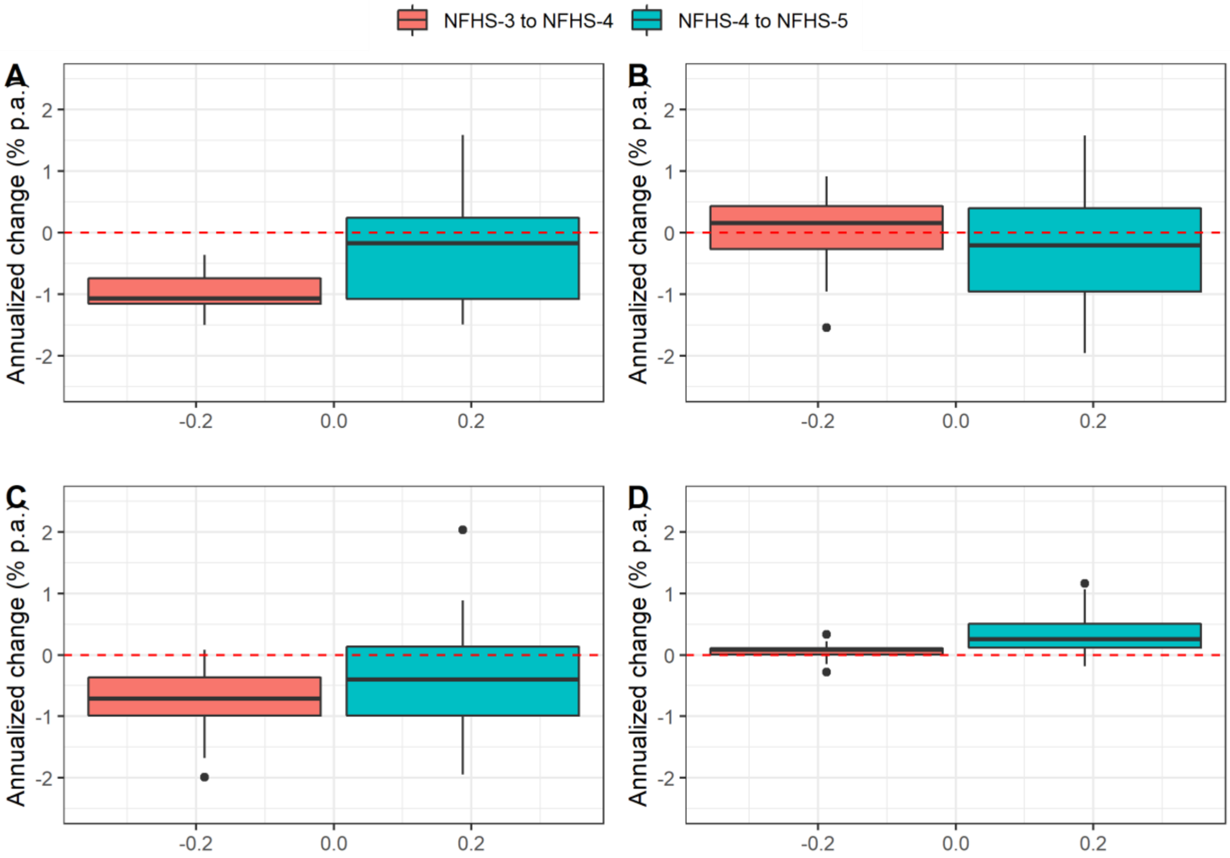

p.a.: percentage points per annum; A: Stunting; B: Wasting; C: Underweight; D: Overweight

Supplement to: Varghese JS, Gupta A, Mehta R, Stein AD, Patel SA. Changes in child undernutrition and overweight in India from 2006 to 2019: an ecological analysis of 36 states. *Glob Health Sci Pract*. 2022;10(5):e2100569. https://doi.org/10.9745/GHSP-D-21-00569

Supplement Figure 4. Scatterplot of annualized changes at state-level between 2006-16 and 2016-21 for development indicators (n = 30)

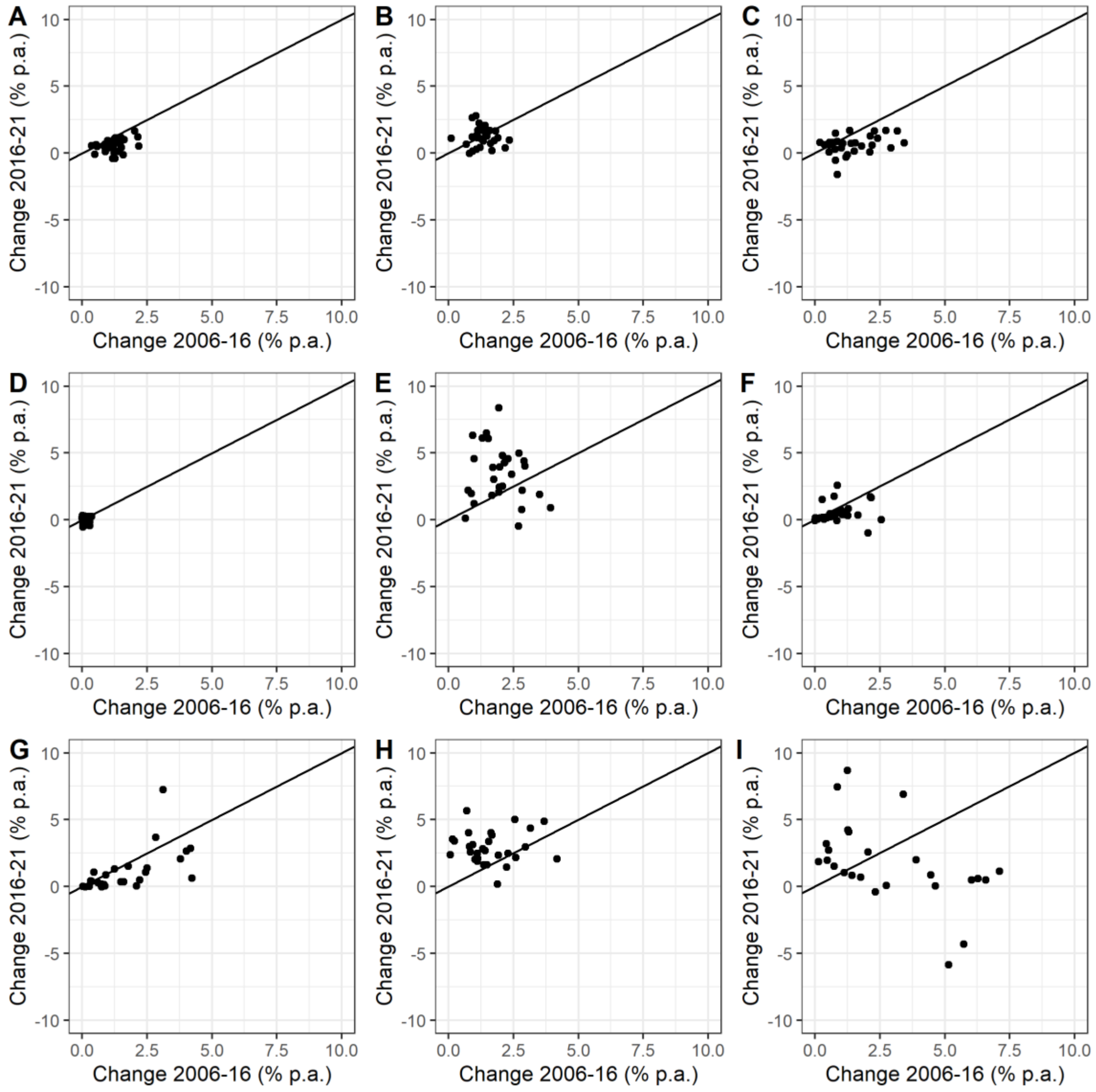

% p.a., annualized percent change (percentage points per annum)
A: Women who are literate (%); B: Women with 10 or more years of schooling; C: Women age 20-24 years first cohabiting or marrying at age 18 years or older (%); D: Female births in last five years (%); E: Population living in households that use an improved sanitation facility; F: Population living in households with an improved drinking-water source (%); G: Population living in households with electricity; H: Households using clean fuel for cooking (%); I: Households with any usual member covered under a health insurance or financing scheme (%)

Supplement to: Varghese JS, Gupta A, Mehta R, Stein AD, Patel SA. Changes in child undernutrition and overweight in India from 2006 to 2019: an ecological analysis of 36 states. *Glob Health Sci Pract*. 2022;10(5):e2100569. https://doi.org/10.9745/GHSP-D-21-00569

Supplement Figure 5. Distribution of annualized change for development indicators (n = 30)

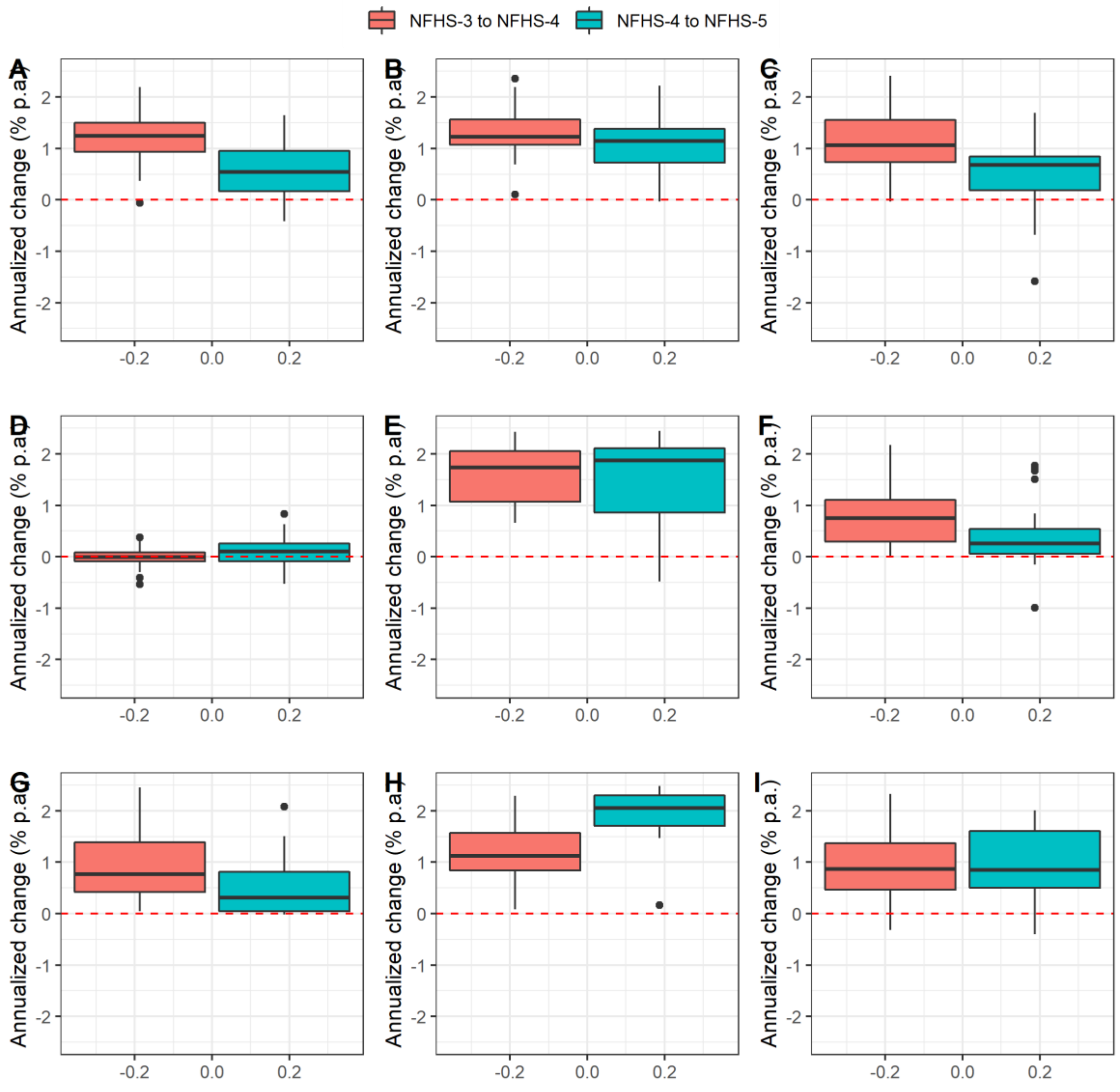

p.a.: percentage points per annum;
A: Women who are literate (%); B: Women with 10 or more years of schooling; C: Women age 20-24 years marrying at age 18 years or older (%); D: Female births in last five years (%); E: Population living in households that use an improved sanitation facility; F: Population living in households with an improved drinking-water source (%); G: Population living in households with electricity; H: Households using clean fuel for cooking (%); I: Households with any usual member covered under a health insurance or financing scheme (%)
